# Oct-4 Expression Maintained Cancer Stem-Like Properties in Lung Cancer-Derived CD133-Positive Cells

**DOI:** 10.1371/journal.pone.0002637

**Published:** 2008-07-09

**Authors:** Yu-Chih Chen, Han-Shui Hsu, Yi-Wei Chen, Tung-Hu Tsai, Chorng-Kuang How, Chien-Ying Wang, Shih-Chieh Hung, Yuh-Lih Chang, Ming-Long Tsai, Yi-Yen Lee, Hung-Hai Ku, Shih-Hwa Chiou

**Affiliations:** 1 Institute of Clinical Medicine, National Yang-Ming University, Taipei, Taiwan; 2 Institute of Traditional Medicine, National Yang-Ming University, Taipei, Taiwan; 3 Institute of Emergency and Critical Care Medicine, National Yang-Ming University, Taipei, Taiwan; 4 Institute of Anatomy and Cell Biology, National Yang-Ming University, Taipei, Taiwan; 5 Division of Thoracic Surgery, Department of Surgery, Taipei Veterans General Hospital and National Yang-Ming University, Taipei, Taiwan; 6 Cancer Center, Taipei Veterans General Hospital and National Yang-Ming University, Taipei, Taiwan; 7 Department of Emergency, Taipei Veterans General Hospital and National Yang-Ming University, Taipei, Taiwan; 8 Department of Medical Research and Education, Taipei Veterans General Hospital and National Yang-Ming University, Taipei, Taiwan; 9 Department of Medical Research and Education, Taipei City Hospital, Taipei, Taiwan; Washington University, United States of America

## Abstract

CD133 (prominin-1), a 5-transmembrane glycoprotein, has recently been considered to be an important marker that represents the subset population of cancer stem-like cells. Herein we report the isolation of CD133-positive cells (LC-CD133^+^) and CD133-negative cells (LC-CD133^−^) from tissue samples of ten patients with non-small cell lung cancer (LC) and five LC cell lines. LC-CD133^+^ displayed higher Oct-4 expressions with the ability to self-renew and may represent a reservoir with proliferative potential for generating lung cancer cells. Furthermore, LC-CD133^+^, unlike LC-CD133^−^, highly co-expressed the multiple drug-resistant marker ABCG2 and showed significant resistance to chemotherapy agents (i.e., cisplatin, etoposide, doxorubicin, and paclitaxel) and radiotherapy. The treatment of Oct-4 siRNA with lentiviral vector can specifically block the capability of LC-CD133^+^ to form spheres and can further facilitate LC-CD133^+^ to differentiate into LC-CD133^−^. In addition, knock-down of Oct-4 expression in LC-CD133^+^ can significantly inhibit the abilities of tumor invasion and colony formation, and increase apoptotic activities of caspase 3 and poly (ADP-ribose) polymerase (PARP). Finally, *in vitro* and *in vivo* studies further confirm that the treatment effect of chemoradiotherapy for LC-CD133^+^ can be improved by the treatment of Oct-4 siRNA. In conclusion, we demonstrated that Oct-4 expression plays a crucial role in maintaining the self-renewing, cancer stem-like, and chemoradioresistant properties of LC-CD133^+^. Future research is warranted regarding the up-regulated expression of Oct-4 in LC-CD133^+^ and malignant lung cancer.

## Introduction

Lung cancer is one of the leading causes of cancer-related deaths in industrialized countries [1], [2]. Radiotherapy and chemotherapy play significant and crucial roles in clinical anti-lung cancer treatment to achieve prolonged patient survival [3], [4]. However, a high failure rate and low median survival rate are observed in patients undergoing chemoradiotherapy with recurrent, intractable lung cancer [5]. To improve the patient survival rate, investigation to elucidate the mechanism of tumorigenesis of lung cancer is needed [5]. Recent data have demonstrated that tumors contain a small subpopulation of cells, i.e., cancer stem-like cells (CSCs) or cancer-initiating cells (CICs), which exhibit a self-renewing capacity and are responsible for tumor maintenance and metastasis [6]. Stem cells have been isolated by their ability to efflux Hoechst 33342 dye and are referred to as the “side population (SP)” [7]. Ho and colleagues isolated and characterized SP cells from six human lung cancer cell lines and showed that an elevated expression of ABCG2 as well as other ATP-binding cassette transporters were positively correlated with resistance to multiple chemotherapeutic drugs [8]. In addition, Gutova and colleagues have purified uPAR-positive CSCs from three lung cancer cell lines. These uPAR-positive cells co-expressed with CD44 and MDR1, and had the ability to promote advanced malignancy and chemoresistance [9].

CD133 (prominin-1), a 5-transmembrane glycoprotein, was first recognized in CD34^+^ progenitor populations from adult blood, bone marrow, and fetal liver cells [10]. Recently, CD133 has been considered an important marker to represent the subset population of CSCs in leukemia, brain tumors, retinoblastoma, renal tumors, pancreatic tumors, colon carcinoma, prostate carcinoma, and hepatocellular carcinoma [11]–[19]. Based on immunohistochemical findings, Hilbe and colleagues suggested that CD133-positive (CD133^+^) progenitor cells play a role in the development of tumor vasculature in patients with non-small-cell lung cancer (NSCLC) [20]. More recently, a well-designed study by Eramo and colleagues showed that lung cancer contains a population of CD133^+^ CSCs able to self-renew and generate an unlimited progeny of non-tumorigenic cells. These CD133^+^ cells are also resistant to conventional chemotherapy [21]. However, the gene regulation mechanisms in maintaining the self-renewal and drug-resistant properties in putative cancer stem-like cells of lung tumors are still unclear.

Oct-4, a member of the family of POU-domain transcription factors, is expressed in pluripotent embryonic stem (ES) and germ cells [22]–[23]. Oct-4 mRNA is normally found in totipotent and pluripotent stem cells of pregastrulation embryos [24]. Knocking out the *Oct-4* gene in mice causes early lethality due to the lack of ICM formation, indicating that Oct-4 has a critical function for self-renewal of ES cells [25]. Oct-4 activates transcription via octamer motifs, and Oct-4 binding sites have been found in various genes, including *fgf 4* (fibroblast growth factor 4) and *pdgf*α*r* (platelet-derived growth factor α receptor) [26], [27]. This suggests that Oct-4 functions as a master switch during differentiation by regulating the pluripotent potentials of the stem cell, and Oct-4 plays a pivotal role in mammalian development [24], [25].

In this study, the CD133-positive cells (LC-CD133^+^) and CD133-negative cells (LC-CD133^−^) were isolated from tissue samples of lung cancer (LC) patients and LC cell lines. These LC-CD133^+^ cells possessed both the characteristics of stem-like cells and malignant tumors. Our data further demonstrated that Oct-4 expression in LC-CD133^+^ is involved in tumor malignancy of lung cancers and exhibits refractory properties for chemoradiotherapy in cancer stem-like cells. These results suggested that the expression of Oct-4 plays a crucial role in maintaining cancer stem-like and chemoradioresistant properties in lung cancer-derived CD133^+^ cells.

## Materials and Methods

### Isolation of CD133^+^ Cell Subset

This research followed the tenets of the Declaration of Helsinki and all samples were obtained after patients provided informed consent. The study was approved by the Institutional Ethics Committee/Institutional Review Board of Taipei Veterans General Hospital. The dissociated cells from the samples of non-small cell lung cancer patients (Table 1) and the lung cancer (LC) cell lines were labeled with 1 mL CD133/l micromagnetic beads per 1 million cells using the CD133 cell isolation kit (Miltenyi Biotech, Auburn, CA). CD133^+^ cells were cultured in a medium consisting of serum-free DMEM/F12 (Gibco-BRL, Gaithersburg, MD), N2 supplement (R&D Systems Inc., Minneapolis), 10 ng/ml human recombinant bFGF (R&D Systems) and 10 ng/ml EGF (R&D Systems) [28].

**Table 1:** The tumor formation abilities of CD133^+^ and CD133^−^ in NSCLC description and tumorigenic characteristics of CD133^+^

					Tumor formation	
No.	Type	Staging/TNM	CD133+(%)	SF	CD133+	CD133−
1	**AC**	IIIB pT4pN3pMX	5.9	Yes	10,000(3/3)	10,000(0/3)
					3,000(3/3)	3,000(0/3)
					1,000(3/3)	1,000(0/3)
2	**AC**	IIIA pT2pN2pMX	2.6	Yes	10,000(3/3)	10,000(0/3)
					3,000(3/3)	3,000(0/3)
					1,000(3/3)	1,000(0/3)
3	**AC**	IIA pT1pN1pMX	0.7	Yes	10,000(3/3)	10,000(0/3)
					3,000(3/3)	3,000(0/3)
					1,000(1/3)	1,000(0/3)
4	**AC**	IIB pT3pN0pMX	1.4	Yes	10,000(3/3)	10,000(0/3)
					3,000(3/3)	3,000(0/3)
					1,000(3/3)	1,000(0/3)
5	**AC**	IIB pT2pN1pMX	0.5	Yes	10,000(3/3)	10,000(0/3)
					3,000(2/3)	3,000(0/3)
					1,000(1/3)	1,000(0/3)
6	**AC**	IIIA pT3pN1pMX	0.7	Yes	10,000(3/3)	10,000(0/3)
					3,000(3/3)	3,000(0/3)
					1,000(1/3)	1,000(0/3)
7	**AC**	IIIB pT3pN3pMX	3.7	Yes	10,000(3/3)	10,000(0/3)
					3,000(3/3)	3,000(0/3)
					1,000(3/3)	1,000(0/3)
8	**SCC**	IA pT1pN0pMX	0.3	Yes	10,000(3/3)	10,000(0/3)
					3,000(2/3)	3,000(0/3)
					1,000(0/3)	1,000(0/3)
9	**SCC**	IIIA pT3pN2pMX	2.3	Yes	10,000(3/3)	10,000(0/3)
					3,000(3/3)	3,000(0/3)
					1,000(2/3)	1,000(0/3)
10	**SCC**	IIIA pT2pN2pMX	1.1	Yes	10,000(3/3)	10,000(0/3)
					3,000(3/3)	3,000(0/3)
					1,000(3/3)	1,000(0/3)

CD133^+^ and CD133^−^ cells were injected into the tail vein of SCID mice, respectively. NSCLC: non-small cell lung cancer. SF: sphere formation. AC: adenocarcinoma. SCC: squamous cell carcinoma. Positive response (Positivity): the tumor formation in the lung tissues of SCID mice. Sphere Formation: Under serum-free medium with bFGF & EGF culture for 4 weeks.

### Cell Viability Determined by Colorimetric Assay

The isolated CD133^+^ and CD133^−^ cells were cultured in a 96-well cell culture cluster (Corning Costar, Acton, MA) at a density of 3×10^3^ cells/well with 100 µl culture medium. At specific time points during cultivation, the medium was discarded and replaced with an equal volume (100 µl) of fresh medium containing 0.2 mg/ml of 3- (4,5-dimethylthiazol-2-yl)-5-(3-carboxymethoxyphenyl)-2-(4-sulfophenyl)-2H-tetrazolium (MTS, Promega, Madison, WI) and 0.038 mg/ml of phenazine methosulfate (PMS; Promega) and incubated for additional 1.5 hours in 37°C 5% CO_2_. Cell viability proportionate to optical density (OD) was measured by colorimetric assay in terms of mitochondria activity to convert tetrazolium salt into a colored soluble formazan product in the medium. The OD values were measured at the wavelength of 490 nm with a 1420 multilabel counter VICTOR from Wallac (PerkinElmer Wallac, Turku, Finland).

### Real-time Reverse Transcription-polymerase Chain Reaction (RT-PCR)

For real-time RT-PCR analysis, the total RNA of cells was extracted by using the RNA_easy_ kit (Qiagen, Valencia, CA). Briefly, the total RNA (1 µg) of each sample was reversely transcribed in 20 µL using 0.5 µg of oligo dT and 200 U Superscript II RT (Invitrogen, Carlsbad, CA). The amplification was carried out in a total volume of 20 µl containing 0.5 µM of each primer, 4 mM MgCl_2_, 2 µl LightCycler FastStart DNA Master SYBR green I (Roche Diagnostics, Pleasanton, CA) and 2 µl of 1∶10 diluted cDNA. The quantification of the unknown samples was performed by LightCycler Relative Quantification Software, version 3.3 (Roche Diagnostics). In each experiment, the GAPDH housekeeping gene was amplified as a reference standard. GAPDH primers were designed: GAPDH(f): GCCAAAAGGGTCATCATC (nt 448–465, GenBank accession no. NM_002046), GAPDH(r): ATGACCTTGCCCACA GCCTT (nt 745–765), Oct-4a(f): CGCAAGCCCTCATTTCAC (nt 5–22, GenBank accession no. NM_002701), Oct-4a(r): CATCACCTCCACCACCTG (nt 98–115, GenBank accession no. NM_002701). PCR reactions were prepared in duplicate and heated to 95°C for 10 minutes followed by 40 cycles of denaturation at 95°C for 10 seconds, annealing at 55°C for 5 seconds, and extension at 72°C for 20 seconds. All PCR reactions were performed in duplicate. Standard curves (cycle threshold values versus template concentration) were prepared for each target gene and for the endogenous reference (GAPDH) in each sample.

### Immunofluorescence Staining for Stem Cell Markers

An avidin-biotin complex method was used for the immunofluorescence staining in the differentiated spheroid and neuronal-like cell. In brief, cells were plated onto poly-L-ornithine-coated glass coverslips and fixed with 4% paraformaldehyde for 15 to 20 minutes at room temperature, and then were washed twice (10 minutes each) with 1× PBS. Cells were permeabilized with 0.1% Triton X-100/PBS for 10 minutes at room temperature, and then twice (10 minutes each) with 1× PBS. The cells were then blocked with blocking solution for 30 minutes and were incubated with primary antibodies (Oct-4, Chemicon, Temecula, CA) for 1 hour at room temperature. We then washed the cells three times (10 minutes each) with 1× PBS. Immunoreactive signals were detected with a mixture of biotinylated rabbit antimouse IgG and Fluorsave (Calbiochem, San Diego, CA). Cells were further probed with fluorescein isothiocyanate (FITC)-tagged secondary antibodies. Fluorescence images were visualized with a fluorescence microscope. To quantitatively analyze the fluorescence intensity, we recorded images with an inverted fluorescence microscope equipped with a CCD camera. The percentage of signal fluorescence per photographed field was analyzed by image processing software (Image Pro-Plus, MediaCybernetics, Inc., Silver Spring, MD).

### FACS Analysis

For cell surface marker identification, a single cell suspension of sixth- to eighth-passage cells from trypsinized spheres was stained with anti-CD133, CD117 (c-Kit), or ABCG2 and secondary fluorescein (FITC)-or phycoerythrin (PE)-coupled antibodies (Dako, Carpinteria). Cells were fixed with 2% paraformaldehyde and were analyzed with a BD FACSCalibur apparatus (Becton, Dickinson and Company, Franklin Lakes, NJ).

### Radiation Treatment for Cell Viability Analysis

The gamma radiation (ionizing irradiation; IR) was delivered by a Theratronic cobalt unit T-1000 (Theratronic International, Inc., Ottawa, Canada) at a dose rate of 1.1Gy/min (SSD = 57.5cm). To evaluate the cell proliferation rate we seeded cells on 24-well plates at a density of 2×10^4^ cells/well. Cells were seeded 24 hours after IR and then they were analyzed by methyle thiazol tetrazolium assay (MTT assay, Sigma-Aldrich, St. Louis, MN). The amount of MTT formazon product was determined by using a microplate reader and the absorbance was measured at 560 nm (SpectraMax 250, Molecular Devices, Sunnyvale, CA).

### Chemotherapeutic Agents

Cisplatin, etoposide (VP16), and paclitaxel were obtained from Sigma-Aldrich and were dissolved in DMSO (Sigma-Aldrich) at 100 mM of stock solution.

### *In Vitro* Cell Invasion Analysis and Soft Agar Colony Assay

The 24-well plate Transwell system with an 8-µm pore size polycarbonate filter membrane (Corning Costar, Corning, NY) was used. The filter membrane was coated with Matrigel (BD Biosciences, San Diego) diluted with serum-free medium and incubated overnight at 37°C. The cell suspensions were seeded to the upper compartment of the Transwell chamber at the cell density of 1×10^5^ in 100 µl serum free medium. After 24 hours, the medium was removed and the filter membrane was fixed with 4% formalin for 1 hour. The opposite surface of the filter membrane facing the lower chamber was stained with Hoechst 33342 (Sigma-Aldrich) for 3 minutes and the migrated cells were then visualized under an inverted microscope. The protocol of soft agar colony assay is described as follows. Each well (35 mm) of a six-well culture dish was coated with 2 ml bottom agar mixture (DMEM, 10% (v/v) FCS, 0.6% (w/v) agar). After the bottom layer solidified, 2 ml top agar-medium mixture (DMEM, 10% (v/v) FCS, 0.3% (w/v) agar) containing 2×10^4^ cells was added, and the dishes were incubated at 37°C for 4 weeks. Plates were stained with 0.5 ml of 0.005% crystal violet for 1 hour and then a dissecting microscope was used to count the number of colonies [29].

### Lentiviral-mediated RNAi

The pLVRNAi vector and pCDH-MCS1-EF1-copGFP vector were purchased from Biosettia Inc. (Biosettia, San Diego, CA). The method of cloning the double-stranded shRNA sequence is described in the manufacturer's protocol. The siRNA oligonucleotide 5′-CCGGCCCTCACTTCACTGCACTGTACTCGAGTACAGTGC AGTGAAGTGAGGGTTTTT-3′ targeting human Oct-4 (NM_002701, nt 1035-1055) was synthesized and cloned into pLVRNAi to generate a lentiviral expression vector. The Oct-4 cDNA was amplified and purified by RT-PCR and cloned into a pCDH-MCS1-EF1-copGFP vector. Lentiviral production was done by transfection of 293T cells using Lipofectamine 2000 (LF2000, Invitrogen). Supernatants were collected 48 hours after transfection and then were filtered; the viral titers were then determined by FACS at 48 hours post-transduction. Subconfluent cells were infected with lentivirus at a multiplicity of infection of 5 in the presence of 8 ìg/ml polybrene (Sigma-Aldrich).

### *In Vivo* Analysis of Tumor Growth and Metastasis

All procedures involving animals were in accordance with the institutional animal welfare guidelines of Taipei Veterans General Hospital. 1000, 3000, and 10^4^ cells were injected into the tail vein of SCID mice and/or nude mice (BALB/c strain) each aged 8 weeks. In vivo GFP imaging was visualized and measured by an illuminating device (LT-9500 Illumatool TLS equipped with excitation illuminating source [470 nm] and filter plate [515 nm]). The tumor size was measured with calipers and the tumor volume was calculated according to the formula (Length×Width^2^)/2. The integrated optical density of green fluorescence intensity was captured and then analyzed by Image Pro-plus software [29].

### Statistical Analysis

Statistical Package of Social Sciences software (version 13.0) (SPSS, Inc., Chicago, IL) was used for statistical analysis. The independent Student's *t*-test or ANOVA was used to compare the continuous variables between groups, whereas the χ^2^ test was applied for comparison of dichotomous variables. The Kaplan-Meier estimate was used for survival analysis, and the log-rank test was used to compare the cumulative survival durations in different patient groups. The level of statistical significance was set at 0.05 for all tests.

## Results

### Isolation and Characterization of Lung Cancer-derived CD133-positive Cells

**Figure 1:**
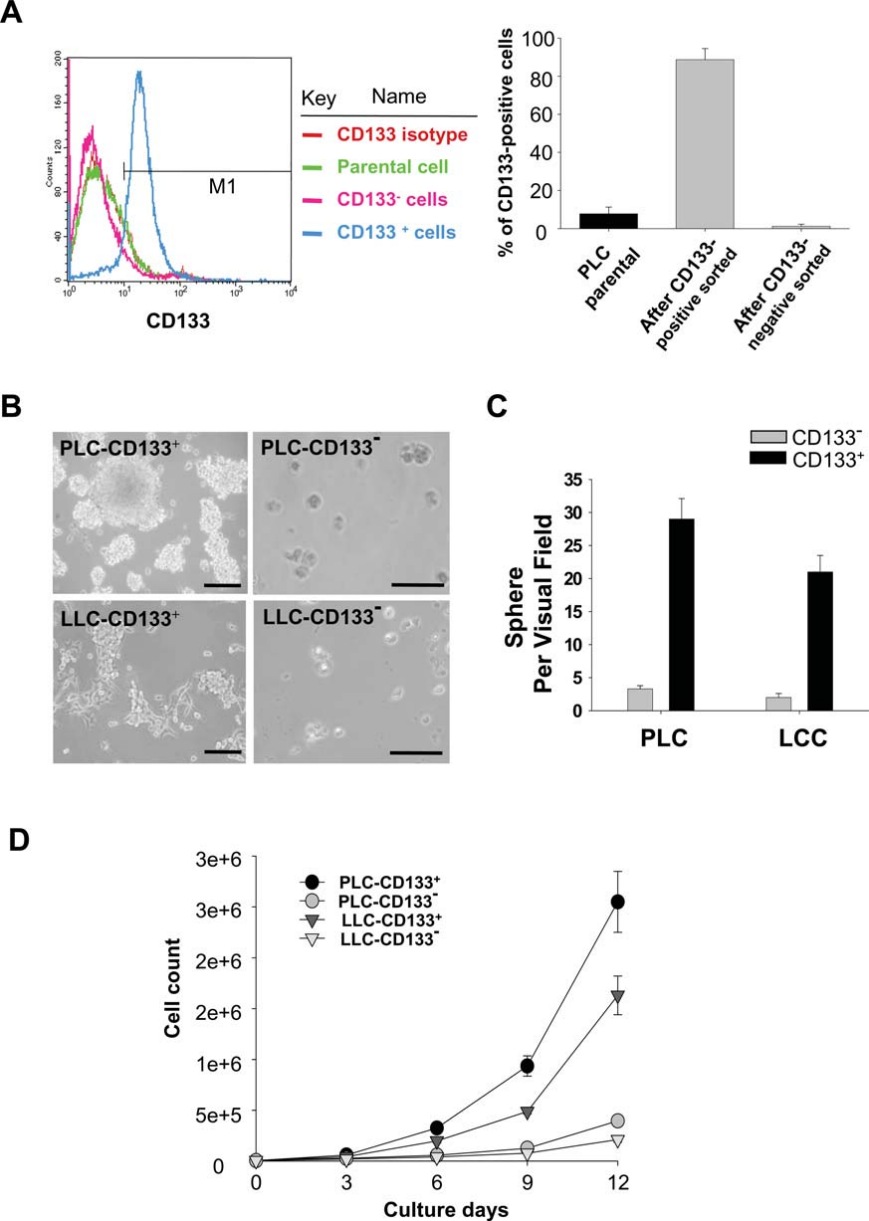
Isolation and characterization of lung cancer-derived CD133^+^ (LC-CD133^+^). (A) Using a magnetic bead method, we sorted CD133^+^ cells from tissue samples of patients with lung cancer (LC), and characterized them by FACS assay. (B) LC-CD133^+^ sorted from two patient with No.1 (PLC-CD133^+^) and No.2 (LLC-CD133^+^) were cultured in bFGF and EGF with DMEM serum-free medium. (C) Evaluation of the formation abilities of spheroid-like bodies (SB) from LC-CD133^+^ and LC-CD133^−^ under serum-free medium with bFGF & EGF. (D) The growth curves of LC-CD133^+^ and LC-CD133^−^ were measured by hemocytometer. Bar: 100 µm. Data shown here are the mean±SD of three experiments.

Using the magnetic bead method, we isolated CD133^+^ cells (Fig. 1A) from tissue samples of ten non-small cell lung cancer (NSCLC) patients (Table 1) and five lung cancer (LC) cell lines (Table S1). The high percentage (97%) of CD133^+^ (LC-CD133^+^) subset was isolated in the LC tissues and parental LC cell line (Fig. 1A). It has been reported that cancer stem-like cells can be cultured in suspension to generate floating spheroid-like bodies (SB) under serum-free medium with bFGF & EGF [30]. We found that LC-CD133^+^ isolated from these ten patients (Table 1) and five LC cell lines (Table S1) can form SB in DF-12 serum-free medium with bFGF and EGF (Fig. 1B; No.1 [PLC] and No.2 [LLC]). Furthermore, the ability to form SB (Fig. 1C) and proliferation rate (Fig. 1D) in LC-CD133^+^ were significantly higher than that in LC-CD133^−^ (p<0.05). In addition, to determine the *in vivo* tumorigenic activity of LC-CD133^+^ and LC-CD133^−^, we injected respective amounts of 1000, 3000, and 10^4^ cells into the tail veins of SCID mice. The results showed that 10^4^ LC-CD133^−^ did not induce tumor formation but 3,000 LC-CD133^+^ from the lung cancer tissues of ten patients and five LC cell lines in xenotransplanted mice can all generate visible tumors 4 weeks after injection (Table 1 and Table S1).

### Increased ABCG2 Expression and Invasive Ability of LC-CD133^+^ *In Vitro*

**Figure 2:**
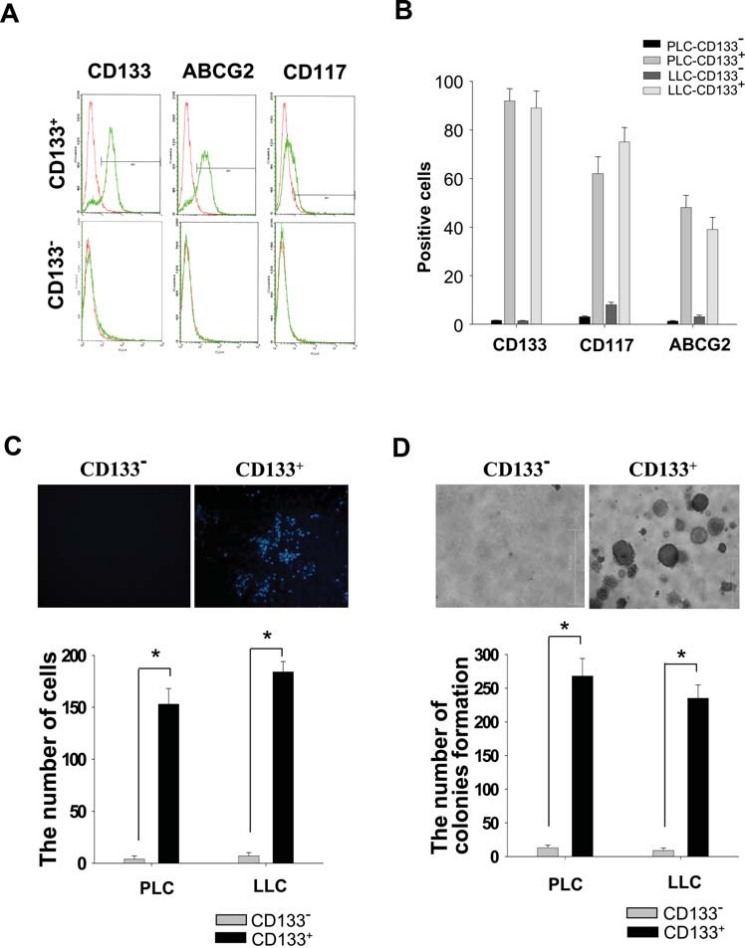
Detection of surface markers and the tumorigenicity in LC-CD133^+^ and LC-CD133^−^ *in vitro*. (A) and (B) The expression levels of CD133, CD117 (c-Kit), and ABCG2 were analyzed by FACS assay in LC-CD133^+^ and LC-CD133^−^. The capabilities of (C) the migration/invasion and (D) the tumor foci (soft agar colony) formation in LC-CD133^+^ were significantly increased compared with LC-CD133^−^ (*p<0.001). Data shown here are the mean±SD of three experiments.

To characterize our isolated LC-CD133^+^, FACS analysis was used to detect the expression profile of cells surface markers. As shown in Figure 2A, the majority of isolated LC-CD133^+^ were stained with higher expression levels of CD133, CD117 (c-Kit), and ABCG2 compared with LC-CD133^−^. This result demonstrated that isolated LC-CD133^+^ were almost ABCG2-positive cells (Fig. 2B). To further evaluate the enhancement of tumorigenicity of LC-CD133^+^, we examined *in vitro* Matrigel-combined Transwell invasion and soft agar colony formation assays. Compared with LC-CD133^−^, LC-CD133^+^ derived from NSCLC Patients No.1 (PLC) and No. 2 (LLC) showed higher invasion activity through Matrigel Transwell invasion assay (*p*<0.001; Fig. 2C). Similarly, the foci formation ability of LC-CD133^+^ from PLC (No.1) and LLC (No.2) was enhanced when compared with the LC-CD133^−^ of those two patients (*p*<0.001; Fig. 2D).

### Increased *In Vivo* Tumor-restoration and Proliferative Ability in LC-CD133^+^

**Figure 3:**
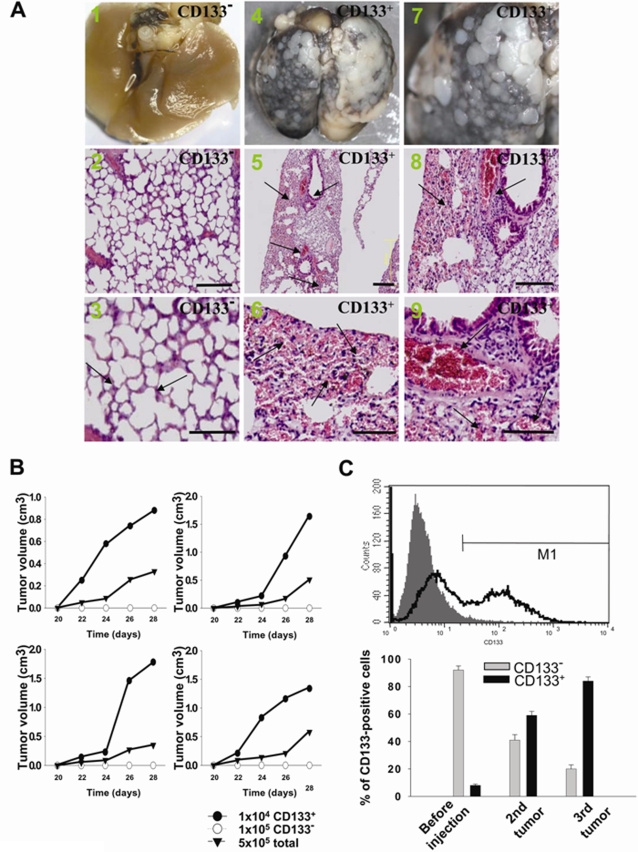
Evaluation of the tumorigenicity of LC-CD133^+^ and LC-CD133^−^ *in vivo*. (A) The i*n vivo* tumorigenicity of LC-CD133^+^ and LC-CD133^−^ in tail vein-injected mice was analyzed by macroscopic and histological examination. A1–3: LC-CD133^−^; arrows: normal alveolar structure of lung. A4–6: LC-CD133^+^; arrows: tumor formation. A7–9: LC-CD133^+^; arrows: neovascularity and thrombosis. Bar: 200 µm. (B) The *in vivo* tumor-restoration and proliferative ability of 10^4^ LC-CD133^+^, 10^5^ LC-CD133^−^ and 5×10^5^ total tumor cells from patient No. 1, 2, 4, and 7 were examined by xenotransplanted tumorigenicity analysis. (C) The tumor repopulation ability of LC-CD133^+^ was studied in transplanted SCID mice. The expression levels of CD133 were determined by FACS analysis from primary LC-CD133^+^, second tumor, and third tumor. Data shown here are the mean±SD of three experiments.

We further evaluated the *in vivo* tumor-restoration and proliferative ability of LC-CD133^+^ and LC-CD133^−^ by xenotransplanted tumorigenicity analysis (Fig. 3A). Four weeks after 10^4^ cells were injected into the tail veins of SCID mice, a significant increase in the multiple nodules of tumor formation on lung surface was noted in the LC-CD133^+^-injected group (Figs. 3A4 & 3A7) but not in the LC-CD133^−^ group (Fig. 3A1). Diffuse infiltrations of LC-CD133^+^ from the lung parenchyma to the alveolar cavity were observed (Figs. 3A5, 3A6, and 3A8). The histological examination demonstrated that the prominent neovascularization and thrombus formation were detected in the pulmonary parenchyma of LC-CD133^+^-injected SCID mice (Fig. 3A9). In contrast, no significant tumor foci or neovascular formation was found in the lungs of LC-CD133^−^-injected SCID mice (Figs. 3A2 & 3A3). We further investigated the *in vivo* tumor growth rate in 10^4^ LC-CD133^+^ cells, 10^6^ LC-CD133^−^ cells, and 5×10^6^ parent tumor cells from the same patient. The finding demonstrated that the tumor growth rate of the 10^4^ LC-CD133^+^ group (from Patients No. 1, 2, 4, and 7; Table 1) was significantly higher than that of the 10^6^ LC-CD133^−^ group and 5×10^6^ parental tumor cell group (Fig. 3B). Furthermore, 10^4^ LC-CD133^+^ isolated from secondary tumors can further generate new (second) tumors from transplanted SCID mice. Results of flow cytometry showed that a high percentage (60%) of CD133-positive cells was detected in the second tumor (Fig. 3C). In addition, one thousand LC-CD133^+^ isolated from the second tumor can also generate a new (third) tumor in transplanted SCID mice (Fig. 3C). To sum, our data indicated that LC-CD133^+^ present self-renewing and repopulation capabilities both *in vitro* and *in vivo*.

### Enhanced Chemo- and Radiation-resistance in LC-CD133^+^

**Figure 4:**
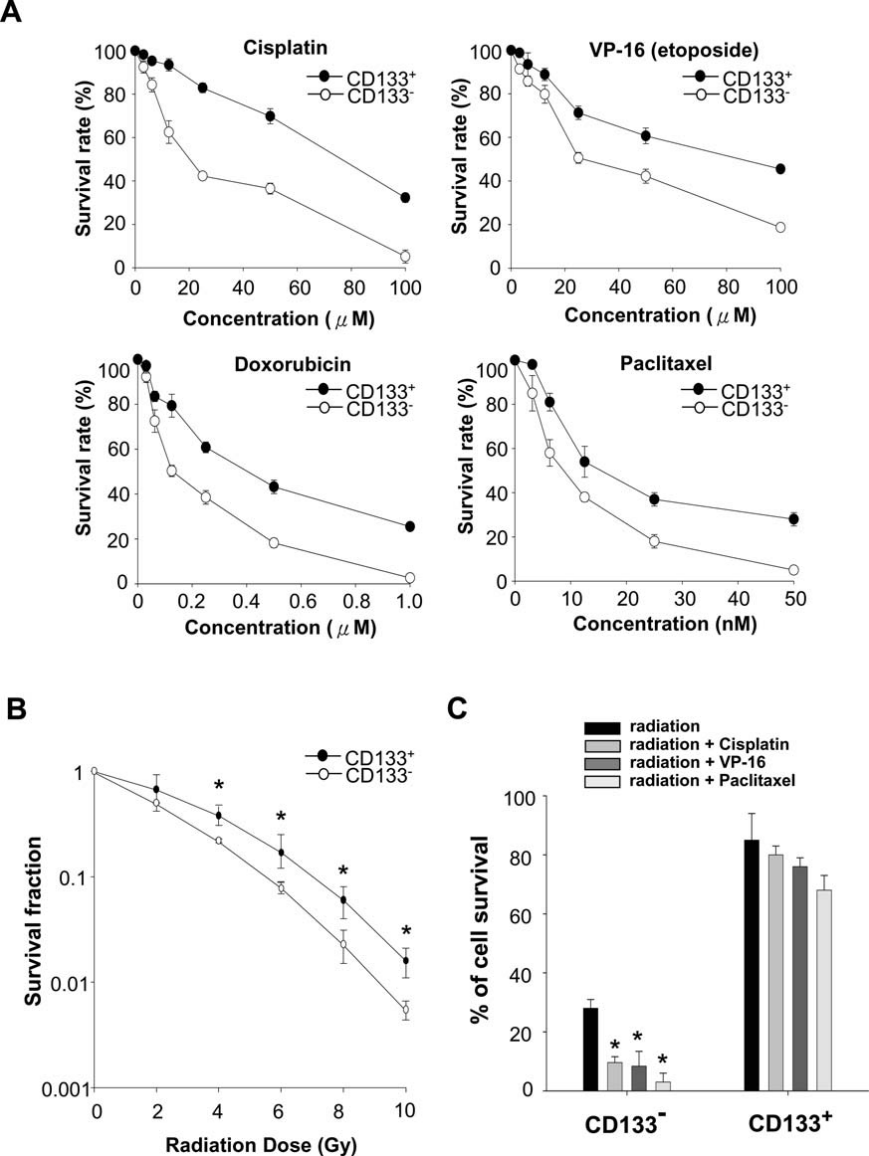
Chemodrugs and radiation sensitivity of LC-CD133^+^ and LC-CD133^−^. (A) Both 10,000 LC-CD133^+^ and LC-CD133^−^ were plated in a 96-well plate and treated with various concentrations of cisplatin, VP-16, doxorubicin, and paclitaxel for 24 hours in 10% FBS/DMEM/F-12 medium. The survival rate was determined by MTT assay. (B) To determine the radiation effect on the tumor growth rate, an ionizing radiation (IR) dose from 0 to 10 Gy was used to treat LC-CD133^+^ and LC-CD133^−^. *p<0.01: LC-CD133^+^ compared with LC-CD133^−^. (C) The combined treatment effect of radiochemotherapy in LC-CD133^+^ and LC-CD133^−^ were further evaluated. The four protocols—radiation (2 Gy) only, radiation with cisplatin (10 µM), radiation with VP-16 (10 µM), and radiation with paclitaxel (10 nM)—were used. *p<0.01. Data shown here are the mean±SD of three experiments.

We evaluated the multidrug (chemotherapy)-resistant abilities of LC-CD133^+^ and LC-CD133^−^. We further tested four common chemotherapeutic agents including cisplatin, VP16 (etoposide), doxorubicin, paclitaxel. Compared with LC-CD133^−,^ LC-CD133^+^ are significantly resistant to the four tested chemotherapeutic agents (*p*<0.01; Fig. 4A). To further determine the radiation effect on the rate of tumor growth, we used an ionizing radiation (IR) dose from 0 to 10 Gy to treat both LC-CD133^+^ and LC-CD133^−^. As shown in Fig. 3B, after IR treatment, the survival rate and number of LC-CD133^+^ were significantly higher than those of LC-CD133^−^ (*p*<0.01). We further found that the LC-CD133^+^ cells possess a higher degree of radioresistance (*p*<0.01; Fig. 4B). Moreover, we investigated the combined treatment effect of radiochemotherapy in LC-CD133^+^. Experiments were conducted with cisplatin (10 µM) alone, VP-16 (10 µM) alone, or combined cisplatin and VP-16 on IR (2 Gy)-treated LC-CD133^+^. As shown in Figure 3C, the data revealed that the cell survival rate in IR-treated LC-CD133^+^ was not significantly decreased by the IR treatment combined with cisplatin, with or without VP-16 (*p*>0.05). On the contrary, cell survival significantly declined after chemotherapy with cisplatin combined with VP-16 in IR-treated LC-CD133^−^ (*p*<0.01; Fig. 4C). These results suggest that LC-CD133^+^ may play a vital role in the tumor's ability to resist radiation and chemotherapies.

### Role of Oct-4 Expression in LC-CD133^+^

**Figure 5:**
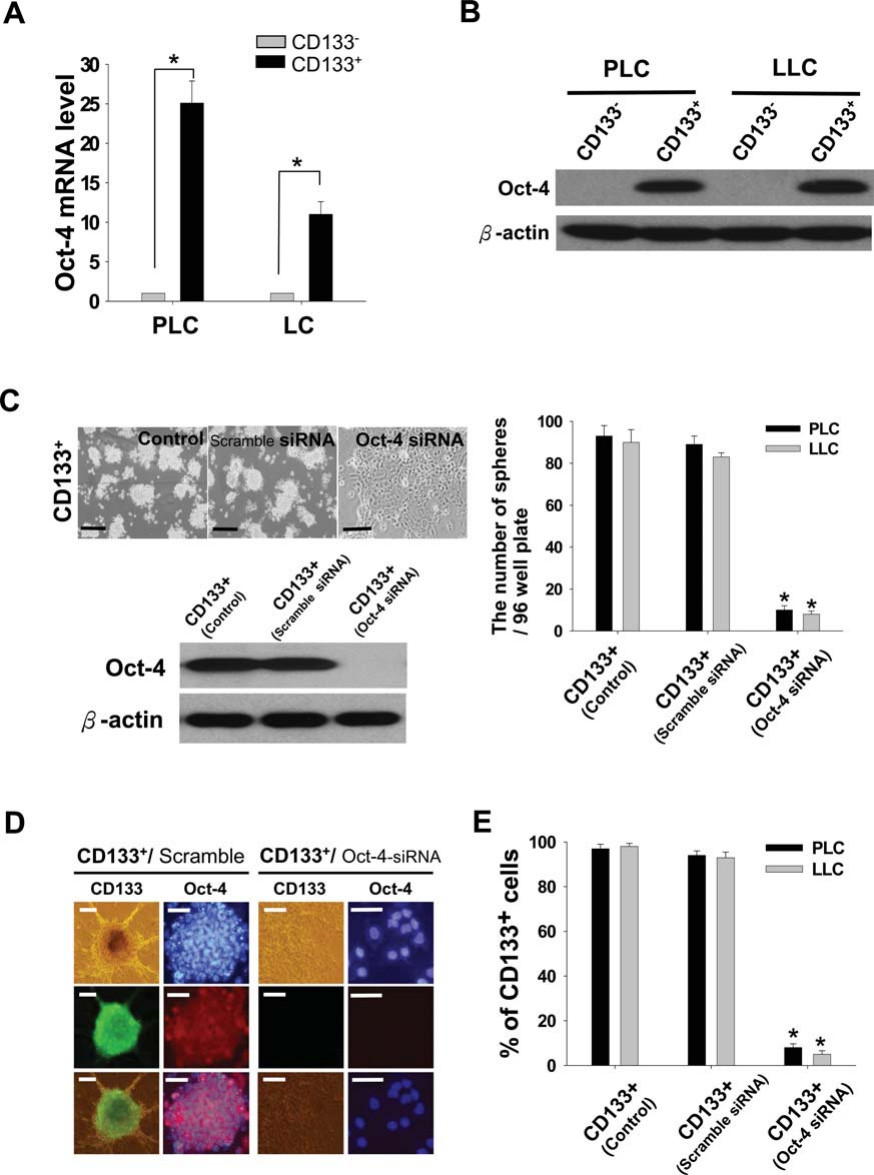
Up-regulated expressions of Oct-4 mRNA and protein in LC-CD133^+^. (A) The amounts of Oct-4 transcripts of isolated LC-CD133^+^ were significantly increased compared with those of LC-CD133^−^ by real-time RT-PCR analyses. (B) Western blott data showed that the protein levels of Oct-4 in LC-CD133^+^ isolated from PLC and LLC were also significantly upregulated compared with those of LC-CD133^−^. (C) The protein expression of Oct-4 in LC-CD133^+^ were effectively blocked by Oct-4 siRNA. Treatment of Oct-4 siRNA in LC-CD133^+^ can impede the capabilities of SB formation and further facilitate SB to differentiate into attached epithelial-like cells. Bar: 100 µm. (D) By using immunofluorescent staining, we showed that the protein expression levels of both Oct-4 and CD133 in LC-CD133^+^ were significantly diminished after Oct-4 siRNA treatment. Bar: 30 µm. (E) The proportion of LC-CD133^−^ were significantly increased in Oct-4 siRNA-treated LC-CD133^+^ by FACS assay. *p<0.001. Data shown here are the mean±SD of three experiments.

Microarray results suggested that the expression level of Oct-4 self-renewal and stemness gene in LC-CD133^+^ was significantly up-regulated than that in LC-CD133^−^. To validate this finding, we examined expression of Oct-4 both transcriptionally and translationally. The amounts of Oct-4 transcript and protein of isolated LC-CD133^+^ (Patients No.1 [PLC] and No.2 [LLC]) were significantly increased compared with those of LC-CD133^−^ by real-time RT-PCR and western blotting analysis (Figs. 5A and 5B). To investigate whether Oct-4 expression plays a role in maintaining self-renewal or cancer stem-like properties in LC-CD133^+^, we used the siRNA method with lentiviral vector for knockdown of Oct-4 expression in LC-CD133^+^. We found it important that the treatment of Oct-4 siRNA in LC-CD133^+^ can significantly interfere with the capabilities of spheroid-like bodies (SB) formation (*p*<0.001; Fig. 5C). After 7 days of the Oct-4 siRNA treatment, the SB of LC-CD133^+^ could not maintain floating spheres but differentiated into attached epithelial-like cells (Fig. 5C). In contrast, the treatment of scramble control siRNA did not influence the SB formation capability in LC-CD133^+^ (Fig. 5C). The SB of LC-CD133^+^ endogenously expressed strong positive signals for Oct-4 and CD133 (Fig. 5D). Furthermore, the immunofluorescent results demonstrated that both the CD133 and Oct-4 expressions in LC-CD133^+^ were significantly blocked after 7 days of Oct-4 siRNA treatment (Fig. 5D). FASC assay confirmed that the amount of CD133 was dramatically decreased in Oct-4 siRNA-treated LC-CD133^+^ and the percentages of LC-CD133^−^ were significantly increased in LC-CD133^+^ after 7 days of Oct-4 siRNA treatment (*p*<0.001; Fig. 5E). These data suggested that Oct-4 may maintain the properties of primitive stem cells and inhibit the tendency for differentiation in LC-CD133^+^.

### Enhanced Chemoradiotherapeutic Sensitivity and Apoptotic Activity in LC-CD133^+^ Treated by Oct-4 siRNA

**Figure 6:**
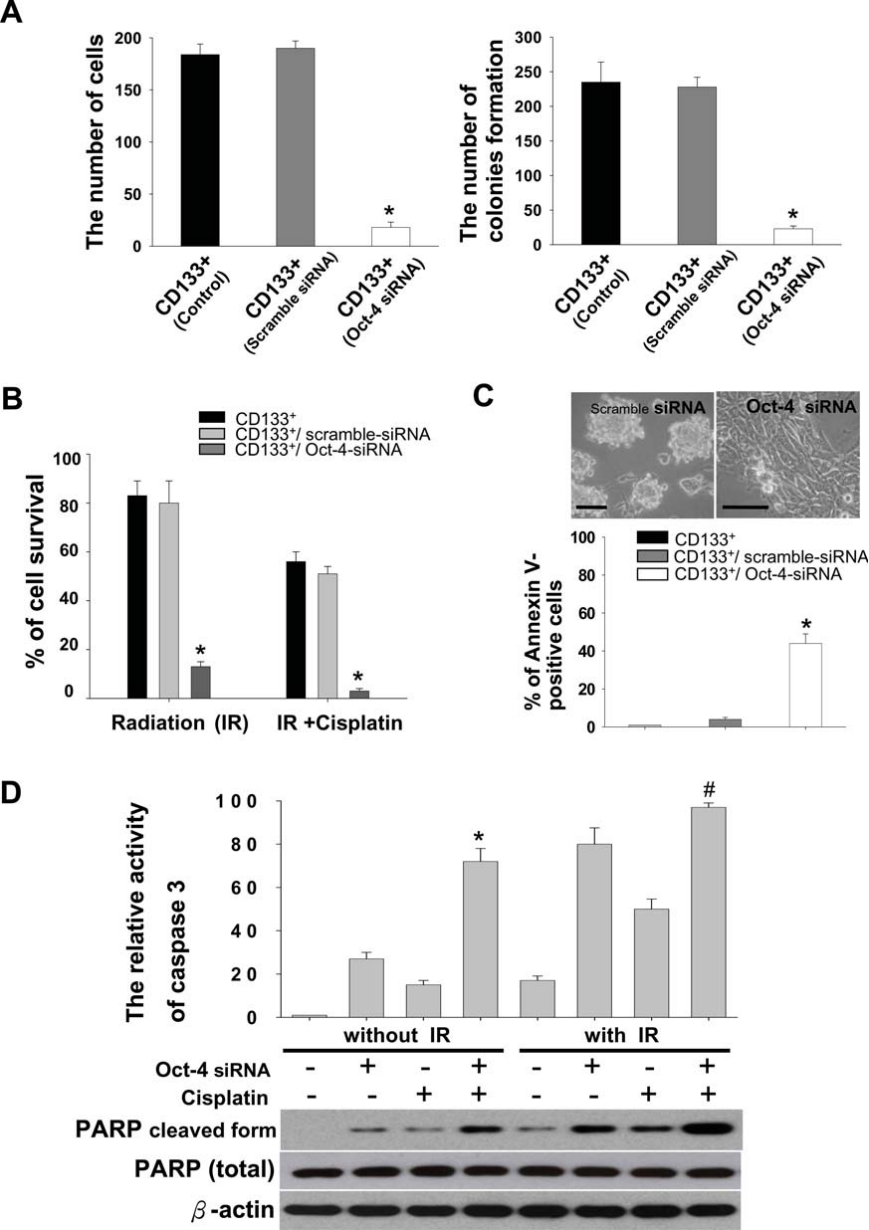
Evaluation of the chemoradiosensitivity and apoptotic activity in the knockdown Oct-4 expression of LC-CD133^+^. (A) Migratory invasion ability and colony formation of LC-CD133^+^ treated by Oct-4 siRNA was significantly decreased compared with non-Oct-4 siRNA-treated LC-CD133^+^ or LC-CD133^+^ treated with scramble-siRNA (control; *p*<0.001). (B) The combined treatment effect of radiochemotherapy in non-Oct-4 siRNA-treated LC-CD133^+^, scramble-siRNA, and Oct-4 siRNA-treated LC-CD133^+^ were further evaluated. Three groups of cells were exposed to IR (2 Gy) only or radiation plus cisplatin. The cell survival rate was determined by MTT assay. After 72 hours of Oct-4 siRNA treatment, (C) the percentage of Annexin V-positive cells and (D) the activities of caspase 3 (detected by ELISA assay) in Oct-4 siRNA-treated LC-CD133^+^ were significantly increased. (D) The western blot data further showed that PARP was significantly induced in Oct-4 siRNA-treated LC-CD133^+^ when exposed to IR alone, cisplatin alone, or IR combined with cisplatin. (*p<0.05: LC-CD133^+^ with Oct-4 siRNA plus cisplatin *vs.* LC-CD133^+^ with Oct-4 siRNA only. #*p*<0.05: LC-CD133^+^ with Oct-4 siRNA plus cisplatin and IR *vs.* LC-CD133^+^ with Oct-4 siRNA plus IR). Bar: 50 µm. Data shown here are the mean±SD of three experiments.

To further study the role of Oct-4 in tumor malignancy of LC-CD133^+^ *in vitro*, the migration/invasive and soft agar colony assay were used. The results showed that the abilities of the *in vitro* migratory invasion and colony formation in LC-CD133^+^ treated by Oct-4 siRNA were significantly decreased compared with non-treated LC-CD133^+^, or LC-CD133^+^ treated with scramble-siRNA (control; *p*<0.001; Fig. 6A). Furthermore, the treatment effect of chemoradiotherapy for the LC-CD133^+^ group can be significantly improved by the treatment of Oct-4 siRNA compared with non-treated LC-CD133^+^ or LC-CD133^+^ treated by scramble-siRNA (Fig. 6B; *p*<0.001). In addition, we found that the apoptotic activities of annexin V (Fig. 6C) and caspase 3 (Fig. 6D; upper part) were quickly and effectively induced in LC-CD133^+^ treated by Oct-4 siRNA after 72 hours. In accordance with the result of cell survival and treatment effects in Oct-4 siRNA-treated LC-CD133^+^ (Fig. 6B), the western blot data further demonstrated that the amounts of activated (cleaved) form of PARP were consistently elevated in LC-CD133^+^ treated by Oct-4 siRNA with IR alone or combined with chemotherapy (Fig. 6D; lower part). Thus, knockdown of Oct-4 expression in LC-CD133^+^ can effectively enhance chemoradiosensitivities and apoptotic activities in response to IR and chemotherapy, suggesting that Oct-4 could be a key factor enables LC-CD133^+^ to resist radiochemotherapeutic stress.

### Inhibition of *In Vivo* Tumorgeneic Potential in Oct-4 siRNA-treated LC-CD133^+^

**Figure 7:**
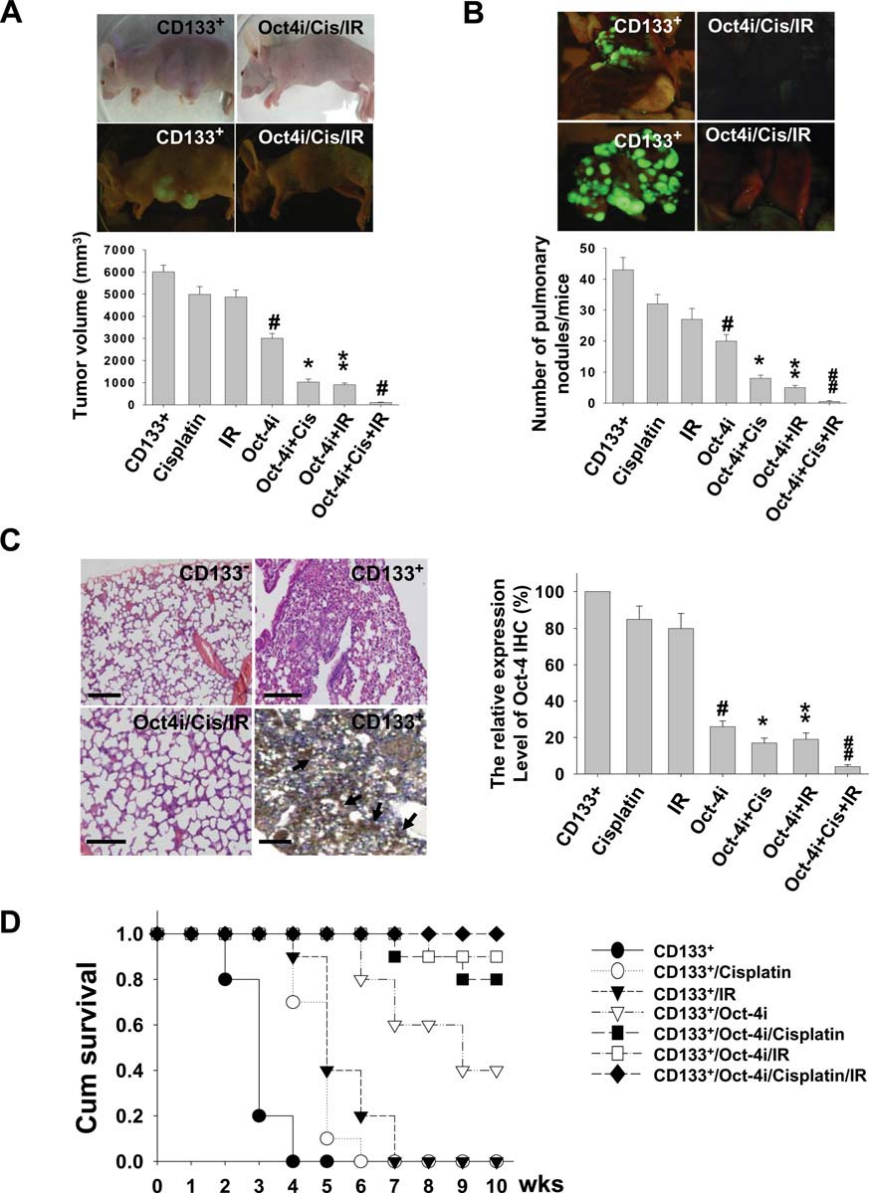
Inhibition of tumorgeneic activity in Oct-4 siRNA-treated LC-CD133^+^ with chemoradiotherapy. LC-CD133^+^-GFP (10^4^) cells of different treatment groups were injected into the subcutaneous sites of nude mice. (A) The tumor volume and (B) The number of pulmonary tumor nodules were significantly decreased in Oct-4 siRNA-treated LC-CD133^+^ when exposed to IR alone, cisplatin alone, or IR combined with cisplatin. Cis: cisplatin; Oct-4i: Oct-4 siRNA. (#*p*<0.01: LC-CD133^+^ treated with Oct-4 siRNA *vs.* LC-CD133^+^. *p<0.01: LC-CD133^+^ treated with Oct-4 siRNA plus cisplatin *vs.* LC-CD133^+^ treated with cisplatin only. **p<0.01: LC-CD133^+^ treated with Oct-4 siRNA plus IR *vs.* LC-CD133^+^ treated with IR only. ##*p*<0.01: LC-CD133^+^ treated with Oct-4 siRNA plus cisplatin and IR *vs.* LC-CD133^+^ with Oct-4 siRNA plus IR). (C) The *in vivo* tumorgeneic activities of LC-CD133^+^- and LC-CD133^−^-injected SCID mice were evaluated by histological review (left upper part). The expression levels of Oct-4 in the lung lesions of LC-CD133^+^-injected SCID mice with the different treatment protocol were studied by immunohistochemistry (IHC). The black arrows indicate the positive signals for Oct-4 expression in the tumors detected by IHC. Bar: 50 µm. (D) Survival analysis of SCID mice injected by LC-CD133^+^, LC-CD133^+^ treated with cisplatin, LC-CD133^+^ treated with IR, and Oct-4 siRNA-treated LC-CD133^+^ groups (IR alone, cisplatin alone, or IR combined with cisplatin). Each group tested six mice (n = 6). Data shown here are the mean±SD of three experiments.

To investigate the treatment effects of chemoradiotherapy on Oct-4 siRNA-treated LC-CD133^+^, LC-CD133^+^ was first transfected by lentivector combined with green fluorescent protein gene (GFP), and then *in vivo* GFP imaging and histological study were used to monitor the tumor-growth effect. We first injected 10^4^ LC-CD133^+^-GFP cells into the subcutaneous sites of nude mice with different treatment protocols. The tumor volumes were significantly decreased in Oct-4 siRNA-treated LC-CD133^+^ when exposed to IR alone, cisplatin alone, or IR combined with cisplatin (*p*<0.01; Fig. 7A). To further evaluate the capabilities of tumor invasion and metastasis of LC-CD133^+^ treated by different regimens, we injected 10^4^ LC-CD133^+^-GFP cells from each treatment groups into the tail vein of SCID mice. The results of *in vivo* GFP imaging showed that the tumor foci of lung and metastatic lesions in the Oct-4-siRNA-treated LC-CD133^+^ groups were significantly lower than those of the LC-CD133^+^ without Oct-4-siRNA-treated groups (*p*<0.01; Fig. 7B). In addition, to investigate the treatment effects of Oct-4 expression in LC-CD133^+^ *in vivo*, we injected the seven groups with different regimens individually into the tail vein of SCID mice for xenotransplanted tumorigenicity analysis (Fig. 7C). Immunohistochemistry (IHC) showed that the expression levels of Oct-4 in the lung tumors of LC-CD133^+^-injected SCID mice were highly expressed in comparison with the other treated-groups (Fig. 7C). Oct-4-IHC levels were significantly decreased in Oct-4 siRNA-treated LC-CD133^+^ when exposed to IR alone, cisplatin alone, or IR combined with cisplatin (*p*<0.01; Fig. 7C). Moreover, using combined chemoradiotherapy with the treatment of Oct-4 siRNA, the mean survival rate of the LC-CD133^+^ group was significantly prolonged compared with the control or other treated groups (*p*<0.05; Fig. 7D). This *in vivo* study also confirmed that the treatment effect of chemoradiotherapy for the LC-CD133^+^ group can be improved by the treatment of Oct-4 siRNA.

## Discussion

Self-renewal and pluripotency are the central features in the definition of embryonic stem cells (ESC), and Oct-4 is a key regulator in this process [24]–[26]. Oct-4 has been suggested as one of four major factors that render the reprogramming capability of adult cells into germline-competent-induced pluripotent stem cells [31]–[33]. Previous studies also showed that mouse pulmonary stem cells endogenously express Oct-4 [34]. Recently, Oct-4 transcript can be consistently detected in human embryonal carcinomas, testicular germ cell tumors, seminomas, and bladder carcinomas [35]–[38]. The expression of Oct-4 has further been shown in human breast cancer stem-like cells, suggesting that its expression may be implicated in self-renewal and tumorigenesis via activating its downstream target genes [39]. Herein we reported the isolation of CD133-positive cells (LC-CD133^+^) from clinical tissue samples and lung cancer cell lines. LC-CD133^+^ showed strong proliferative and invasive capabilities *in vitro* and *in vivo* (Figs. 1, 2, and 3). LC-CD133^+^ also displayed significant resistance to chemotherapeutic agents (Fig. 4). We also demonstrated that Oct-4 expression was transcriptionally and translationally up-regulated in LC-CD133^+^ (Fig. 5). Indeed, Oct-4 functions as a master switch during differentiation by regulating the pluripotent potential in stem cells [31]–[33]. Using the siRNA method with lentiviral vector for knockdown of Oct-4 expression in LC-CD133^+^, our data showed that the treatment of Oct-4 siRNA can block the sphere formation of LC-CD133^+^ and further facilitate LC-CD133^+^ to differentiate into LC-CD133^−^ (Fig. 5). Furthermore, *in vivo* animal studies demonstrated the IHC of Oct-4 in the lung tumors of LC-CD133^+^-injected SCID mice were prominently up-regulated, and the total lung tumor volume as well as Oct-4 IHC levels can be significantly decreased in LC-CD133^+^-injected mice by the treatment of Oct-4 siRNA with or without chemoradiotherapy (Fig. 7). In addition, we showed that increased incidence of Oct-4 expression correlated positively with the advanced stage of 78 lung cancers (Figs. S1A–C). To our knowledge, this is the first study to show that Oct-4 expression plays a crucial role in maintaining self-renewal and cancer stem-like properties in LC-CD133^+^.

The property of resistance to chemotherapy and irradiation treatment is the major clinical criterion to characterize “cancer stem-like cells (CSCs)” [6]. The existence of cancer stem-like cells may explain why conventional anti-cancer therapies are able only to suppress or shrink a tumor but often cannot completely eradicate it, resulting in eventual recurrence [6], [40], [41]. Consistent with these hypotheses, LC-CD133^+^ were significantly resistant to cisplatin, VP16 (eptoposide), doxorubicin, and paclitaxel than LC-CD133^−^ (*p*<0.001; Fig. 4). Even IR alone or a single chemodrug can effectively inhibit cell growth of LC-CD133^−^ (Fig. 4); however, IR treatment combined with cisplatin and VP-16 still failed to cause cell death in treated LC-CD133^+^ (Fig. 4D). To overcome resistance to radiotherapy and chemotherapy in LC-CD133^+^, treatment of Oct-4 siRNA was used and results showed that the knockdown Oct-4 in LC-CD133^+^ can significantly improve the anti-cancer effect in single- or combination-treated LC-CD133^+^ *in vitro* and *in vivo* (Figs. 6 and 7). Moreover, the mean survival rate of the LC-CD133^+^ group can be significantly prolonged after the treatment of Oct-4 siRNA with IR and chemotherapy (Fig. 7). Recently, Oct-4 has been suggested to be a protector for survival of ES cells from apoptosis induced by etoposide, UV, or heat shock through the Stat3/Survivin pathway [42]. Consistent with this important finding, our results suggest that knockdown of Oct-4 expression can effectively enhance the chemoradiosensitivity of LC-CD133^+^ through activating the apoptotic activities of caspase 3 and PARP (Fig. 6). Importantly, our *in vivo* animal study and clinical data provide the evidence that the amount of Oct-4 in LC-CD133^+^ (Fig. 7C) and in patients with high-grade lung cancer (Fig. S1) is positively correlated with the degree of resistance to chemoradiation therapy. Taken together, these results indicate that the up-regulated expression of Oct-4 in LC-CD133^+^ may contribute to the development of chemoradioresistance in patients with lung cancer.

Recent studies have revealed that the human ABCG2 transporter is a P-glycoprotein that causes multidrug resistance (MDR) including mitoxantrone, doxorubicin, and topoisomerase I inhibitors of irinotecan, topotecan, and 7-ethyl-10-hydroxycamptothecin (topoisomerase inhibitor) and gefitinib (an inhibitor of EGF receptor) in patients with lung cancer [41], [43]. Hirschmann-Jax and colleagues were the first to observe that “side population” cancer stem-like cells isolated from cell lines and patients with neuroblastoma expressed high levels of ABCG2 and ABCG3 transporter genes as well as a greater capacity to expel cytotoxic drugs [44]. Monzani and colleagues further showed that cancer stem-like cells derived from the melanoma cell line highly co-expressed CD133 and ABCG2 markers with enhanced tumorigenic potential [45]. In this study, we found that LC-CD133^+^ are highly co-expressed with ABCG2 transporter and are significantly resistant to conventional treatment methods compared with LC-CD133^−^ (Figs. 2 & 4). Interestingly, a significant down-regulating of ABCG2 expression and an increase in the chemosensitivity of LC-CD133^+^ were observed when the Oct-4 siRNA treatment was given (Data not shown). Thus, more studies are needed to investigate whether over-expression of Oct-4, CD133, and/or ABCG2 play a role in the development of MDR in LC-CD133^+^ or surrogate markers of therapeutic response in patients with lung cancer.

In conclusion, we demonstrated that LC-CD133^+^ display a higher Oct-4 expression with the ability to self-renew and may represent a reservoir with unlimited proliferative potentials for generating lung cancer cells. The resistance of LC-CD133^+^ to *in vitro* and *in vivo* chemoradiotherapy is partially due to preferential activation of Oct-4 gene expression. In addition, these data support that the up-regulated expressions of the Oct-4, self-renewing gene of embryonic stem cells, play an important role in the tumorigenicity of patients with lung cancer.

## Supporting Information

Table S1 (0.05 MB DOC)

Figure S1 Correlation of Oct-4 expression levels and the clinical grading and survival rate in the patients with lung cancers. (A) Detection of Oct-4 expression in 78 NSCLC lung cancer patients with different stages by using immunohistochemistry (IHC). Black arrows: positive signals for Oct-4 by IHC. Bar: 50 um. (B) Oct-4 was detected in the almost high grades (III & IV) of lung cancer tissues, and significantly expressed the higher intensity of Oct-4-positive signals in grade III & IV (high grade) than those in grade I & II (low grade) lung cancers patients (p<0.01). (C) Kaplan-Meier analysis of overall survival in 78 lung cancer patients with or without Oct-4 expression.
(7.61 MB TIF)
