# Supplementary material for: Oct-4 Expression Maintained Cancer Stem-Like Properties in Lung Cancer-Derived CD133-Positive Cells
Source: PLoS One. 2008 Jul 9;3(7):e2637. doi: 10.1371/journal.pone.0002637 (PMC2440807; doi:10.1371/journal.pone.0002637)
Supplement: Table S1 — (0.05 MB DOC) [file pone.0002637.s001.doc]

**Table S1.**

The tumor formation abilities of CD133^+^ and CD133^–^ derived from human lung cancer cell line

|  |  |  |  | **Tumor formation** | |
| --- | --- | --- | --- | --- | --- |
| **No.** | **Type** | **CD133+(%)** | **SF** | **CD133**+ | **CD133–** |
| **A549** | **NSCLC** | 0.8 | Yes | 10,000(3/3) | 10,000(0/3) |
|  |  |  |  | 3,000(3/3) | 3,000(0/3) |
|  |  |  |  | 1,000(2/3) | 1,000(0/3) |
| **H1299** | **NSCLC** | 0.7 | Yes | 10,000(3/3) | 10,000(0/3) |
|  |  |  |  | 3,000(3/3) | 3,000(0/3) |
|  |  |  |  | 1,000(1/3) | 1,000(0/3) |
| **CCL-1** | **NSCLC** | 0.4 | Yes | 10,000(3/3) | 10,000(0/3) |
|  |  |  |  | 3,000(3/3) | 3,000(0/3) |
|  |  |  |  | 1,000(1/3) | 1,000(0/3) |
| **CCL-5** | **NSCLC** | 1.1 | Yes | 10,000(3/3) | 10,000(0/3) |
|  |  |  |  | 3,000(3/3) | 3,000(0/3) |
|  |  |  |  | 1,000(2/3) | 1,000(0/3) |
| **C299** | **NSCLC** | 0.3 | Yes | 10,000(3/3) | 10,000(0/3) |
|  |  |  |  | 3,000(2/3) | 3,000(0/3) |
|  |  |  |  | 1,000(0/3) | 1,000(0/3) |

CD133^+^ and CD133^–^ cells were injected into the tail vein of SCID mice, respectively. NSCLC: non-small cell lung cancer. SF: sphere formation. Positive response (Positivity): the tumor formation in the lung tissues of SCID mice. Sphere Formation: Under serum-free medium with bFGF & EGF culture for 4 weeks. A549, HEL299, CCL-1, CCL-5, and C299 are all human NSCLC lung cancer cell lines.
